# Supplementary material for: Collective interactions among organometallics are exotic bonds hidden on lab shelves
Source: Nat Commun. 2022 Apr 19;13:2069. doi: 10.1038/s41467-022-29504-0 (PMC9018958; doi:10.1038/s41467-022-29504-0)
Supplement: Supplementary file 1 — Supplementary Information [file 41467_2022_29504_MOESM1_ESM.pdf]

Supplementary Information: Collective Interactions Among Organometallics Are Exotic Bonds Hidden on Lab Shelves Cina Foroutan-Nejad et al.

Shahin Sowlati-Hashjin,[1] Vojtech Sadek,[2,3] SeyedAbdolreza Sadjadi,[4] Mikko Karttunen,[5,6,7] Angel Martin-Pendas,[8]* Cina Foroutan-Nejad[9]*

1. Institute of Biomedical Engineering, University of Toronto, Toronto, ON M5S 3G9, Canada.
2. CEITEC − Central European Institute of Technology, Masaryk University, Kamenice 5, CZ-62500 Brno, Czechia.
3. Department of Chemistry, Faculty of Science, Masaryk University, Kamenice 5, CZ-62500 Brno, Czechia.
4. Department of Physics, Faculty of Science, Laboratory for Space Research, The University of Hong Kong, Hong Kong SAR, China.
5. Department of Chemistry, The University of Western Ontario, 1151 Richmond Street, London, Ontario, Canada N6A 3K7.
6. Department of Physics and Astronomy, The University of Western Ontario, 1151 Richmond Street, London, Ontario N6A 5B7, Canada.
7. Centre for Advanced Materials and Biomaterials Research, The University of Western Ontario, 1151 Richmond Street, London, Ontario, Canada N6K 3K7.
8. Departamento de Química Física y Analítica, University of Oviedo, 33006 Oviedo, Spain.
10. Institute of Organic Chemistry, Polish Academy of Sciences, Kasprzaka 44/52, 01-224 Warsaw, Poland.

–

Corresponding author's emails:

AMP: ampendas@uniovi.es

CFN: cforoutan-nejad@icho.edu.pl

Supplementary Table 1. The dissociation energies defined as the energy differences between relaxed fragments and complexes, D_0, deformation, E_{Def}, and promotion energies, E_{Pro}, in kcal.mol^{-1}. All energies are computed for dissociation of the complexes to a neutral AX_3 and an anionic AX_3 at DFT level. Lower dissociation energies for dissociation to a neutral AX_3 in all cases suggest that in gas-phase the species tend to follow this reaction path. Furthermore, negative promotion energies in anionic dissociation suggests instability of the chosen fragment compared to those in neutral pathway. On the other hand, lower deformation energies for the anionic dissociation prove that the AX_3 species are closer to an anionic form in their complexes.

**Dissociation**	**Anionic AX_3**			**Neutral AX_3**		
**Molecules**	**D_0**	**E_{Def}**	**E_{Pro}**	**D_0**	**E_{Def}**	**E_{Pro}**
**$LiBH_3^-$**	-35.1	0.4	41.3	-22.3	3.2	51.3
**$NaBH_3^-$**	-34.9	0.3	12.4	-18.0	2.0	27.5
**KBH_3^-**	-33.0	0.2	3.9	-15.8	0.3	20.9
**$MgBH_3$**	-193.6	0.2	-83.8	-8.5	0.7	100.8
**$CaBH_3$**	-161.5	0.1	-36.7	-11.7	0.5	112.8
**$LiAlH_3^-$**	-30.6	0.2	83.3	-28.3	12.6	73.2
**$NaAlH_3^-$**	-29.6	0.6	21.8	-23.2	9.7	19.0
**$KAlH_3^-$**	-26.4	1.3	4.4	-19.7	6.9	5.5
**$MgAlH_3$**	-180.4	4.3	-145.7	-5.8	0.4	32.8
**$CaAlH_3$**	-146.2	4.0	-95.5	-6.8	0.7	47.3
***i*-$CaAlH_3$ [a,b,d]**	-155.0	22.1	53.8	-15.6	75.5	139.8
**$LiCF_3$**	-152.3	3.5	10.5	-61.7	14.8	89.7
**$NaCF_3$**	-132.6	3.2	-0.1	-48.6	15.4	71.7
**$BeCF_3^+$**	-440.6	26.1	-98.8	-52.1	0.5	315.4
**$MgCF_3^+$ [c]**	-344.9	23.3	-157.6	-30.5	1.0	179.1
***i*-$LiCF_3$ [b,c,d]**	-153.4	2.8	16.9	-62.8	52.0	58.3
***i*-$NaCF_3$ [b,c,d]**	-134.0	1.2	18.7	-50.0	44.4	59.4
***i*-KCF_3 [b,d]**	-118.7	0.7	27.5	-54.5	41.9	50.6
***i*-$MgCF_3^+$ [b,d]**	-332.5	5.6	21.3	-18.1	62.8	278.5
***i*-$CaCF_3^+$ [b,d]**	-282.7	8.4	17.6	-44.8	70.1	193.7
***i*-$SrCF_3^+$ [b,d]**	-263.6	4.6	31.9	-44.5	59.4	196.2
**$MgC(CN)_3^+$ [b]**	-285.5	49.0	27.1	-33.5	50.6	277.4
**$CaC(CN)_3^+$ [b]**	-243.9	29.2	5.9	-68.4	27.9	182.6
**$SrC(CN)_3^+$ [b]**	-227.9	24.0	14.4	-71.2	23.0	172.1
**$LiCH_3$**	-181.8	0.2	10.3	-50.3	9.7	132.3
**$NaCH_3$**	-160.6	0.4	-18.4	-35.7	7.7	99.2
**KCH_3**	-135.8	0.2	-1.2	-30.7	9.1	95.1
**$BeCH_3^+$**	-514.2	0.7	-57.3	-84.7	5.8	367.1
**$MgCH_3^+$**	-406.4	1.4	-175.6	-51.2	3.8	177.3
**$CaCH_3^+$**	-328.4	0.2	-62.6	-49.6	8.9	207.5
**$SrCH_3^+$**	-307.5	0.2	-52.1	-47.5	9.3	198.8
**$LiC(Ph)_3$**	-135.9	4.1	30.1	-45.3	4.8	120.0
**$NaC(Ph)_3$**	-116.6	2.7	27.5	-32.6	4.1	110.1
**$KC(Ph)_3$**	-105.9	1.8	31.9	-41.6	3.4	94.6
**$MgC(Ph)_3^+$**	-357.1	9.5	-181.9	-42.7	7.7	134.2
**$CaC(Ph)_3^+$**	-307.2	8.0	1.1	-69.3	8.1	238.9
**$SrC(Ph)_3^+$**	-285.6	5.9	8.7	-66.4	6.1	227.6
**$LiC(CH_3)_3$ [c]**	-167.0	0.9	13.0	-33.6	11.5	135.8
**$NaC(CH_3)_3$ [c]**	-147.3	0.9	-17.7	-20.5	9.8	100.3
**$KC(CH_3)_3$ [c]**	-123.2	0.6	2.8	-16.1	11.1	99.3
**$MgC(CH_3)_3^+$**	-403.8	6.7	-238.5	-46.6	15.2	110.2
**$CaC(CH_3)_3^+$**	-316.4	2.6	-66.1	-35.7	7.4	209.8
***i*-$LiC(CH_3)_3$ [d]**	-160.7	5.6	6.6	-27.3	7.7	137.9
***i*-$NaC(CH_3)_3$ [b,d]**	-131.5	6.9	-2.8	-4.7	6.7	124.1
***i*-$KC(CH_3)_3$ [b,d]**	-115.4	9.9	12.4	-8.4	5.4	124.0
***i*-$BeC(CH_3)_3^+$ [c,d]**	-520.8	-1.1	-26.7	-89.5	3.3	400.2
***i*-$MgC(CH_3)_3^+$ [b,d]**	-388.3	27.1	-359.8	-31.1	9.6	15.0
***i*-$CaC(CH_3)_3^+$ [c,d]**	-320.1	13.8	-69.9	-39.4	6.3	218.3
***i*-$SrC(CH_3)_3^+$ [e,d]**	-298.9	15.1	-57.0	-36.9	5.5	214.5

[a]. Data are obtained from the BS-DFT calculation.

[b]. The metal forms a multicenter bond within the context of QTAIM.

[c]. The global minimum of the molecule.

[d]. i- represents inverted structures.

[e]. The global minimum of the molecule has a C_S point group, 1.6 kcal.mol^{-1} lower in energy; therefore, it is not discussed here.

Supplementary Table 2. The magnitudes of V_{XC}(A, B) and V_C(A, B) sorted from the most to the least stabilizing for M–A bonds in group **1** and **2** molecules as well as for specified bonds in our test set. To assess the bond type, *i.e.*, covalency-ionicity, the V_{XC}(A, B) values are used. From N_2 (V_{xc} = –586.4 kcal.mol^{-1}) to SeSe bond in H_2Se_2 (V_{xc} = –94.4 kcal.mol^{-1}) is chosen as the domain of covalent bonds. The conventionally pure ionic bonds from LiF/KCl with V_{XC} = –31.0 kcal.mol^{-1} to virtually zero is marked by a blue line. Besides, the border between the stabilizing and destabilizing V_C(A, B) values are marked by a black line. The red, blue, and black lines are shown in **Figure 1** as well.

V_{XC}(A,B)	Bond	V_{XC}(A,B)	Bond	V_C(A,B)	Bond	V_C(A,B)	Bond
-586.4	N_2	-47.2	$CaCl_2$	-851.0	CO	-27.1	*i*-$LiC(CH_3)_3$
-499.0	NO^+	-45.8	$CaC(CH_3)_3^+$	-699.1	BeO	-22.7	$KC(Ph)_3$
-481.4	CC[a]	-44.8	BeF_2	-669.0	CN^-	-16.4	*i*-$KC(CH_3)_3$
-448.8	NO	-42.1	Na_2O	-637.7	BF_3	-15.2	SeH[a]
-427.3	O_2	-42.1	MgF_2	-490.5	NB[d]	-9.6	*i*-$NaC(CH_3)_3$
-374.1	CN^-	-41.6	$MgCl_2$	-476.9	$BeCH_3^+$	-0.9	$KAlH_3^-$
-368.6	NO^-	-41.4	$BeCl_2$	-399.2	BeF_2	1.1	*i*-$MgC(CH_3)_3^+$
-330.4	CC[b]	-40.1	$NaCH_3$	-396.9	BCl_3	1.4	H_2S
-296.2	CO	-39.1	$NaAlH_3^-$	-368.9	BH[d]	3.5	$NaAlH_3^-$
-226.8	F_2	-38.76	$NaC(CH_3)_3$	-355.4	$BeCl_2$	6.9	SH[a]
-224.9	OO[a]	-38.1	KF	-341.1	MgO	7.0	SeSe[a]
-219.9	NN[b]	-36.6	Li_2O	-340.9	CaO	13.8	KBH_3^-
-195.0	Cl_2	-35.3	KCH_3	-310.5	PH_3	15.7	CC[c]
-189.6	CC[c]	-33.6	$MgBH_3$	-310.1	MgF_2	19.3	$NaBH_3^-$
-179.9	CH[b]	-32.8	$SrC(Ph)_3^+$	-306.7	NO^+	20.8	CH[c]
-179.9	CH[a]	-32.2	$KC(CH_3)_3$	-254.9	$MgCl_2$	20.8	SS[a]
-179.1	H_2S	-31.2	LiF	-242.5	CaF_2	21.3	Br_2
-177.3	SH[a]	-31.0	KCl	-216.2	H_2O	22.0	H_2Se
-177.1	CH[c]	-29.8	$CaC(Ph)_3^+$	-205.4	K_2O	22.6	NO^-
-172.5	SS[a]	-28.3	NaCl	-201.3	$CaCH_3^+$	23.6	$LiAlH_3^-$
-166.5	NH_3	-27.8	$NaCF_3$	-198.3	$CaCl_2$	23.6	CH[b]
-165.2	Br_2	-25.5	*i*-$BeC(CH_3)_3^+$	-196.6	$MgCH_3^+$	23.9	H_2
-165.2	NH[b]	-25.2	$LiCH_3$	-183.5	$SrCH_3^+$	26.3	CH[a]
-164.9	H_2	-25.0	$CaAlH_3$	-174.5	LiF	28.9	Cl_2
-161.0	SeH[a]	-24.8	$LiC(CH_3)_3$	-153.6	$CaC(CH_3)_3^+$	29.4	$SrC(CN)_3^+$
-156.6	NH[d]	-24.6	$CaBH_3$	-148.9	NH_4^+	37.1	F_2
-154.3	CaO	-24.3	LiCl	-146.1	LiCl	39.5	$CaC(CN)_3^+$
-152.9	H_2Se	-24.1	$KAlH_3^-$	-141.8	$LiCH_3$	41.5	$MgAlH_3$
-138.43	NH_4^+	-21.3	$LiCF_3$	-134.0	OH[a]	41.9	$LiBH_3^-$
-130.86	PH_3	-20.8	$LiBH_3^-$	-132.8	KF	42.1	CC[b]
-124.3	H_2O	-18.7	$NaBH_3^-$	-130.6	*i*-$BeC(CH_3)_3$	43.4	OO[a]
-119.0	OH[a]	-18.6	KCF_3	-130.2	$MgC(CH_3)_3^+$	63.0	$MgC(CN)_3^+$
-110.4	MgO	-17.9	$MgAlH_3$	-125.9	$LiC(CH_3)_3$	67.1	NN[b]
-99.81	BeO	-14.1	*i*-$LiC(CH_3)_3$	-120.9	$MgC(Ph)_3^+$	71.6	$CaAlH_3$
-94.4	SeSe[a]	-13.7	KBH_3^-	-116.7	NaCl	80.2	CC[a]
-89.4	BCl_3	-13.7	*i*-$KC(CH_3)_3$	-114.8	NH[d]	84.1	$LiCF_3$
-89.1	BH[d]	-11.3	$CaAlH_3$	-104.7	KCl	93.8	$NaCF_3$
-78.9	$MgCH_3^+$	-10.8	$KC(Ph)_3$	-86.3	$NaCH_3$	98.8	O_2
-72.2	$MgC(CH_3)_3^+$	-10.69	$LiC(Ph)_3$	-81.8	NH_3	100.5	KCF_3
-71.9	$SrCH_3^+$	-10.55	$SrC(CN)_3^+$	-77.8	KCH_3	114.3	$MgBH_3$
-71.5	$CaC(CH_3)_3^+$	-10.14	$CaC(CN)_3^+$	-77.8	Li_2O	133.9	N_2
-71.1	$CaCH_3^+$	-9.3	*i*-$NaC(CH_3)_3$	-74.3	*i*-$SrC(CH_3)_3^+$	149.3	$BeCF_3^+$
-68.5	$BeCH_3^+$	-8.8	$NaC(Ph)_3$	-68.3	$CaC(CH_3)_3^+$	157.8	$CaBH_3$
-63.3	BF_3	-7.1	*i*-$MgC(CH_3)_3^+$	-67.6	NO	168	*i*-KCF_3
-62.9	$MgCF_3^+$	-2.6	$MgC(CN)_3^+$	-65.8	$NaC(CH_3)_3$	188.8	*i*-$NaCF_3$
-61.6	$BeCF_3^+$	-2.3	*i*-$MgCF_3^+$	-65.4	$CaC(Ph)_3^+$	223.2	*i*-$LiCF_3$
-56.3	$MgC(Ph)_3^+$	-2.1	*i*-$CaCF_3^+$	-64.5	$SrC(Ph)_3^+$	163.7	$MgCF_3^+$
-56.2	NB[d]	-1.8	*i*-$SrCF_3^+$	-58.8	$KC(CH_3)_3$	239.5	$CaAlH_3$
-54.0	CaF_2	-0.9	*i*-$NaCF_3$	-58.4	Na_2O	338.3	*i*-$SrCF_3^+$
-53.1	*i*-$SrC(CH_3)_3^+$	-0.9	*i*-KCF_3	-49.6	$LiC(Ph)_3$	351.1	*i*-$CaCF_3^+$
-49.3	K_2O	0.6	*i*-$LiCF_3$	-49.1	NH[b]	403.1	*i*-$MgCF_3^+$
-48.3	$LiAlH_3^-$			-29.0	$NaC(Ph)_3$		

[a] in H_2X_2 (X = O, S, Se)
[b] in H_4X_2 (X = C, N)
[c] in C_2H_6
[d] in NH_3BH_3

Supplementary Table 3. Atomic charges of M and A in MAX_3 systems, Q(M) and Q(A), the interatomic exchange-correlation and Coulombic energy components of IQA interaction energy between metal and Al, B, or central C atoms in the studied systems, V_{XC}(M, A) and V_C(M, A), and those of the metals with the substituents on the central atom, V_{XC}(M, X) and V_C(M, X), in kcal.mol^{-1}. All data are computed at CASSCF level.

Molecules	Angle	Q(M)	Q(A)	V_{XC}(M,A)	V_{XC}(M,T)	V_C(M,A)	V_C(M,T)	V_{Int}(M,A)	V_{Int}(M,T)	ICI_{xc}	ICI_C
**$LiBH_3^-$** [a]	7.61	-0.007	1.561	-16.7	-29.9	44.2	-39.4	27.4	-69.3	0.559	-1.122
**$NaBH_3^-$** [a]	5.38	-0.161	1.756	-12.4	-29.8	27.4	-13.2	15.0	-42.9	0.417	-2.081
**KBH_3^-** [a]	3.52	-0.685	2.046	-3.8	-23.5	-46.1	11.1	-49.9	-12.4	0.160	-4.161
**$MgBH_3$**	3.13	0.450	1.891	-20.7	-53.1	151.3	-33.9	130.6	-87.1	0.390	-4.458
**$CaBH_3$** [a]	2.40	0.596	1.842	-17.2	-46.6	171.2	-55.9	154.0	-102.6	0.368	-3.061
**$NaAlH_3^-$** [a]	13.51	-0.103	1.687	-25.1	-30.9	9.4	-12.2	-15.7	-43.2	0.811	-0.768
**$KAlH_3^-$** [a]	12.21	-0.140	1.764	-19.8	-26.2	10.1	-7.1	-9.7	-33.3	0.755	-1.428

Supplementary Table 4. Atomic charges of M and A in MAX_3 systems, Q(M) and Q(A), the interatomic exchange-correlation and Coulombic energy components of IQA interaction energy between metal and Al, B, or central C atoms in the studied systems, V_{XC}(M, A) and V_C(M, A), and those of the metals with the substituents on the central atom, V_{XC}(M, X) and V_C(M, X), in kcal.mol^{-1}. All data are computed at CCSD level.

Molecules	Angle	Q(M)	Q(A)	V_{XC}(M,A)	V_{XC}(M,T)	V_C(M,A)	V_C(M,T)	V_{Int}(M,A)	V_{Int}(M,T)	ICI_{xc}	ICI_C
**$LiBH_3^-$** [a]	7.61	-0.041	1.548	-22.7	-40.0	37.5	-31.8	14.8	-71.8	0.569	-1.178
**$NaBH_3^-$** [a]	5.38	-0.231	1.767	-14.2	-36.9	14.1	-4.4	-0.1	-41.3	0.384	-3.214
**KBH_3^-** [a]	3.52	-0.353	1.862	-10.0	-32.8	-2.5	2.8	-12.5	-30.0	0.305	-0.882
**$MgBH_3$**	3.13	0.415	1.844	-21.6	-55.0	137.3	-28.7	115.7	-83.8	0.393	-4.782
**$CaBH_3$** [a]	2.40	0.499	1.843	-16.4	-49.2	151.2	-37.7	134.8	-86.9	0.334	-4.011
**$NaAlH_3^-$** [a]	13.51	-0.169	1.732	-30.9	-40.5	1.1	-1.1	-29.7	-41.6	0.762	-1.006
**$KAlH_3^-$** [a]	12.21	-0.221	1.811	-23.5	-33.7	-2.8	1.8	-26.3	-31.8	0.697	-1.526

Supplementary Table 5. Atomic charges of M and A in MAX_3 systems, Q(M) and Q(A), the interatomic exchange-correlation and Coulombic energy components of IQA interaction energy between metal and Al, B, or central C atoms in the studied systems, V_{XC}(M, A) and V_C(M, A), and those of the metals with the substituents on the central atom, V_{XC}(M, X) and V_C(M, X), in kcal.mol^{-1}. All data are computed at DFT-CCSD level.

Molecules	Q(M)	Q(A)	V_{XC}(M,A)	V_{XC}(M,T)	V_C(M,A)	V_C(M,T)	V_{Int}(M,A)	V_{Int}(M,T)	ICI_{xc}	ICI_C
**$LiBH_3^-$** [a]	0.007	1.243	-36.8	-55.6	28.4	-39.1	-8.4	-94.7	0.662	-0.726
**$NaBH_3^-$** [a]	-0.301	1.565	-24.5	-49.5	-5.3	3.2	-29.7	-46.3	0.494	-1.656
**KBH_3^-** [a]	-0.467	1.717	-12.9	-35.9	-24.9	9.6	-37.8	-26.3	0.358	-2.594
**$MgBH_3$**	0.395	1.664	-28.4	-60.8	112.0	-26.6	83.7	-87.4	0.466	-4.211
**$CaBH_3$** [a]	0.497	1.641	-24.0	-55.9	128.5	-42.2	104.5	-98.1	0.429	-3.045
**$NaAlH_3^-$** [a]	-0.236	1.673	-39.3	-51.0	12.3	5.8	-51.6	-45.3	0.771	2.121
**$KAlH_3^-$** [a]	-0.300	1.756	-29.8	-41.6	-18.5	8.1	-48.4	-33.5	0.716	-2.284

Supplementary Table 6. Fuzzy atom partitioning: atomic charges of M and A in MAX_3 systems, Q(M) and Q(A), the interatomic exchange-correlation and Coulombic energy components of IQA interaction energy between metal and Al, B, or central C atoms in the studied systems, $V_{XC}(M, A)$ and $V_C(M, A)$, and those of the metals with the substituents on the central atom, $V_{XC}(M, X)$ and $V_C(M, X)$, in kcal.mol^{-1}.

Molecules	Q(M)	Q(A)	V_{XC}(M,A)	V_C(M,A)	V_{Int}(M,A)	V_{XC}(M,T)	V_C(M,T)	V_{Int}(M,T)	ICI_{XC}	ICI_C
**$LiBH_3^-$**	-0.291	1.462	-49.0	-10.7	-59.8	-99.3	4.6	-94.7	0.494	-2.322
**$NaBH_3^-$**	-0.432	1.590	-51.1	-44.8	-95.9	-111.0	11.5	-99.4	0.460	-3.881
**KBH_3^-**	-0.408	1.655	-37.2	-36.8	-74.0	-90.5	9.3	-81.2	0.411	-3.951
**$MgBH_3$**	0.230	1.584	-94.2	49.1	-45.0	-190.6	-9.9	-200.5	0.494	-4.944
**$CaBH_3$**	0.332	1.613	-74.1	71.8	-2.3	-181.8	-16.7	-198.6	0.408	-4.292
**$LiAlH_3^-$**	0.021	1.071	-114.7	3.8	-110.8	-135.0	-22.8	-157.8	0.849	-0.168
**$NaAlH_3^-$**	-0.241	1.330	-98.8	-24.6	-123.4	-122.2	-1.0	-123.2	0.808	25.471
**$KAlH_3^-$**	-0.277	1.432	-67.3	-25.4	-92.7	-86.7	-2.3	-89.1	0.776	10.868
**$MgAlH_3$**	-0.018	1.781	-62.5	-33.7	-96.2	-135.2	-11.3	-146.5	0.462	2.984
**$CaAlH_3$**	0.263	1.632	-75.5	30.6	-44.9	-121.4	-11.7	-133.1	0.622	-2.619
***i*-$CaAlH_3$** [a,b,d]	1.087	1.334	-21.3	228.9	207.6	-178.2	-208.4	-386.6	0.120	-1.098
**$LiCF_3$**	0.718	1.435	-59.1	97.7	38.7	-71.9	-82.2	-154.2	0.821	-1.189
**$NaCF_3$**	0.657	1.600	-75.2	81.2	6.0	-94.1	-65.2	-159.3	0.799	-1.246
**$BeCF_3^+$**	1.306	1.466	-103.7	191.5	87.8	-129.2	-125.9	-255.2	0.802	-1.520
**$MgCF_3$** [c]	1.180	1.763	-102.4	204.1	101.7	-137.9	-60.0	-197.9	0.743	-3.400
***i*-$LiCF_3$** [b,c,d]	0.870	1.826	-0.8	280.0	279.2	-64.7	-126.7	-191.4	0.013	-2.209
***i*-$NaCF_3$** [b,c,d]	0.902	1.847	-1.1	243.3	242.2	-88.4	-127.2	-215.6	0.012	-1.913
***i*-KCF_3** [b,d]	0.979	1.838	-0.9	233.8	232.8	-102.4	-140.6	-243.1	0.009	-1.663
***i*-$MgCF_3^+$** [b,d]	1.740	1.748	-2.6	495.7	493.1	-150.3	-285.4	-435.7	0.017	-1.737
***i*-$CaCF_3^+$** [b,d]	1.807	1.739	-2.2	451.4	449.2	-185.3	-276.4	-461.6	0.012	-1.634
***i*-$SrCF_3^+$** [b,d]	3.356	1.731	-0.6	859.4	858.9	-471.9	-2613.7	-3085.6	0.001	-0.329
**$MgC(CN)_3^+$** [b]	1.199	0.584	-24.6	72.8	48.2	-247.6	-119.3	-366.9	0.099	-0.610
**$CaC(CN)_3^+$** [b]	1.041	0.447	-12.6	61.5	48.9	-265.3	-3.1	-268.4	0.048	-19.992
**$SrC(CN)_3^+$** [b]	2.317	0.434	-7.0	132.1	125.1	-188.5	-653.0	-841.5	0.037	-0.202
**$LiCH_3$**	0.721	-0.291	-61.5	-75.6	-137.1	-68.1	-107.5	-175.6	0.903	0.703
**$NaCH_3$**	0.619	-0.173	-83.7	-52.1	-135.9	-93.4	-78.4	-171.8	0.896	0.665
**KCH_3**	0.733	-0.163	-82.5	-52.9	-135.4	-91.6	-90.3	-181.8	0.901	0.587
**$BeCH_3^+$**	1.430	-0.562	-116.0	-274.2	-390.2	-127.0	-236.3	-363.3	0.914	1.160
**$MgCH_3^+$**	1.267	-0.327	-121.7	-143.6	-265.3	-134.3	-127.1	-261.4	0.906	1.130
**$CaCH_3^+$**	1.500	-0.283	-135.4	-141.1	-276.6	-150.1	-170.3	-320.4	0.902	0.829
**$SrCH_3^+$**	1.863	-0.435	-106.0	-273.3	-379.3	-116.4	-375.7	-492.1	0.911	0.728
**$LiC(Ph)_3$**	-0.700	-0.378	-54.7	35.9	-18.8	-428.6	-184.2	-612.8	0.128	-0.195
**$NaC(Ph)_3$**	-0.447	-0.192	-46.8	11.3	-35.5	-365.2	-106.8	-471.9	0.128	-0.106
**$KC(Ph)_3$**	-0.190	-0.102	-43.1	1.7	-41.4	-301.5	-51.0	-352.5	0.143	-0.033
**$MgC(Ph)_3^+$**	0.472	-0.522	-97.3	-78.8	-176.1	-225.8	-20.5	-246.3	0.431	3.841
**$CaC(Ph)_3^+$**	-0.400	-0.198	-87.1	2.1	-85.0	-584.2	-212.1	-796.3	0.149	-0.010
**$SrC(Ph)_3^+$**	3.190	-0.589	-149.5	-803.3	-952.9	-431.2	-2227.1	-2658.2	0.347	0.361
**$LiC(CH_3)_3$** [c]	0.629	-0.477	-64.8	-85.4	-150.2	-81.7	-81.1	-162.8	0.793	1.053
**$NaC(CH_3)_3$** [c]	0.488	-0.297	-84.0	-51.5	-135.5	-108.6	-52.5	-161.0	0.773	0.982
**$KC(CH_3)_3$** [c]	0.640	-0.211	-79.5	-49.5	-129.0	-101.5	-65.6	-167.1	0.783	0.755
**$BeC(CH_3)_3^+$**	1.170	-0.696	-112.8	-252.9	-365.6	-137.3	-157.0	-294.4	0.821	1.611
**$MgC(CH_3)_3^+$**	0.893	-0.536	-109.7	-126.7	-236.4	-150.0	-49.6	-199.7	0.731	2.553
**$CaC(CH_3)_3^+$**	1.241	-0.405	-140.0	-138.4	-278.4	-190.5	-103.2	-293.8	0.735	1.341
***i*-$LiC(CH_3)_3$** [d]	-0.414	-0.306	-68.6	17.0	-51.6	-352.4	-94.3	-446.7	0.195	-0.180
***i*-$NaC(CH_3)_3$** [b,d]	0.597	-0.128	-19.8	-13.5	-33.3	-105.6	-55.2	-160.9	0.187	0.244
***i*-$KC(CH_3)_3$** [b,d]	0.664	-0.089	-28.0	-10.8	-38.8	-123.6	-60.5	-184.1	0.227	0.178
***i*-$BeC(CH_3)_3^+$** [c,d]	0.037	-0.235	-86.6	-24.1	-110.7	-470.4	1.2	-469.3	0.184	-20.836
***i*-$MgC(CH_3)_3^+$** [b,d]	0.353	-0.036	-7.1	-0.7	-7.7	-53.1	13.6	-39.5	0.133	-0.049
***i*-$CaC(CH_3)_3^+$** [c,d]	0.592	-0.180	-104.3	-30.7	-135.0	-337.8	-2.5	-340.3	0.309	12.155
***i*-$SrC(CH_3)_3^+$** [e,d]	3.417	-0.735	-190.6	-1151.3	-1341.9	-531.0	-2839.6	-3370.5	0.359	0.405

[a]. Data are obtained from the BS-DFT calculation.

[b]. The metal forms a multicenter bond within the context of QTAIM.

[c]. The global minimum of the molecule.

[d]. i- represents inverted structures.

[e]. The global minimum of the molecule has a C_S point group, 1.6 kcal.mol^{-1} lower in energy; therefore, it is not discussed here.

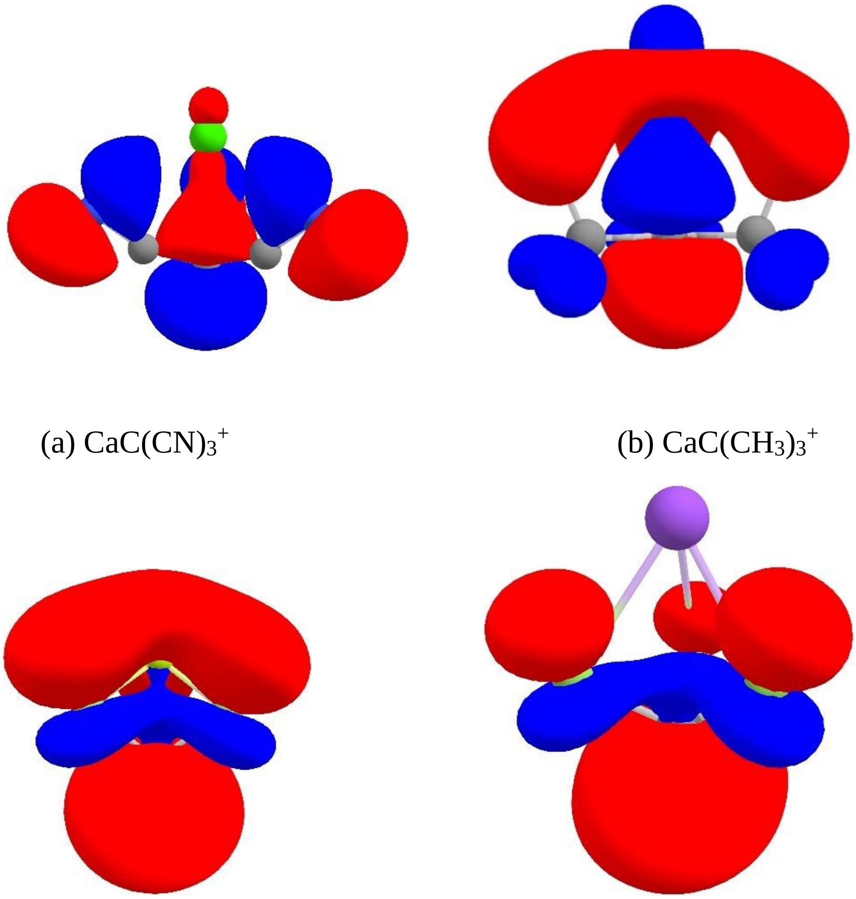

(a) $CaC(CN)_3^+$ (b) $CaC(CH_3)_3^+$

(c) *i*-$BeCF_3^+$ (d) *i*-$NaCF_3$

Supplementary Figure 1. Examples of HOMOs for systems with (a) 2e-2c bonds between M and C, (b) and (c) 2e-multicenter MOs, and (d) nonbonding MOs between M and AX_3 fragments.

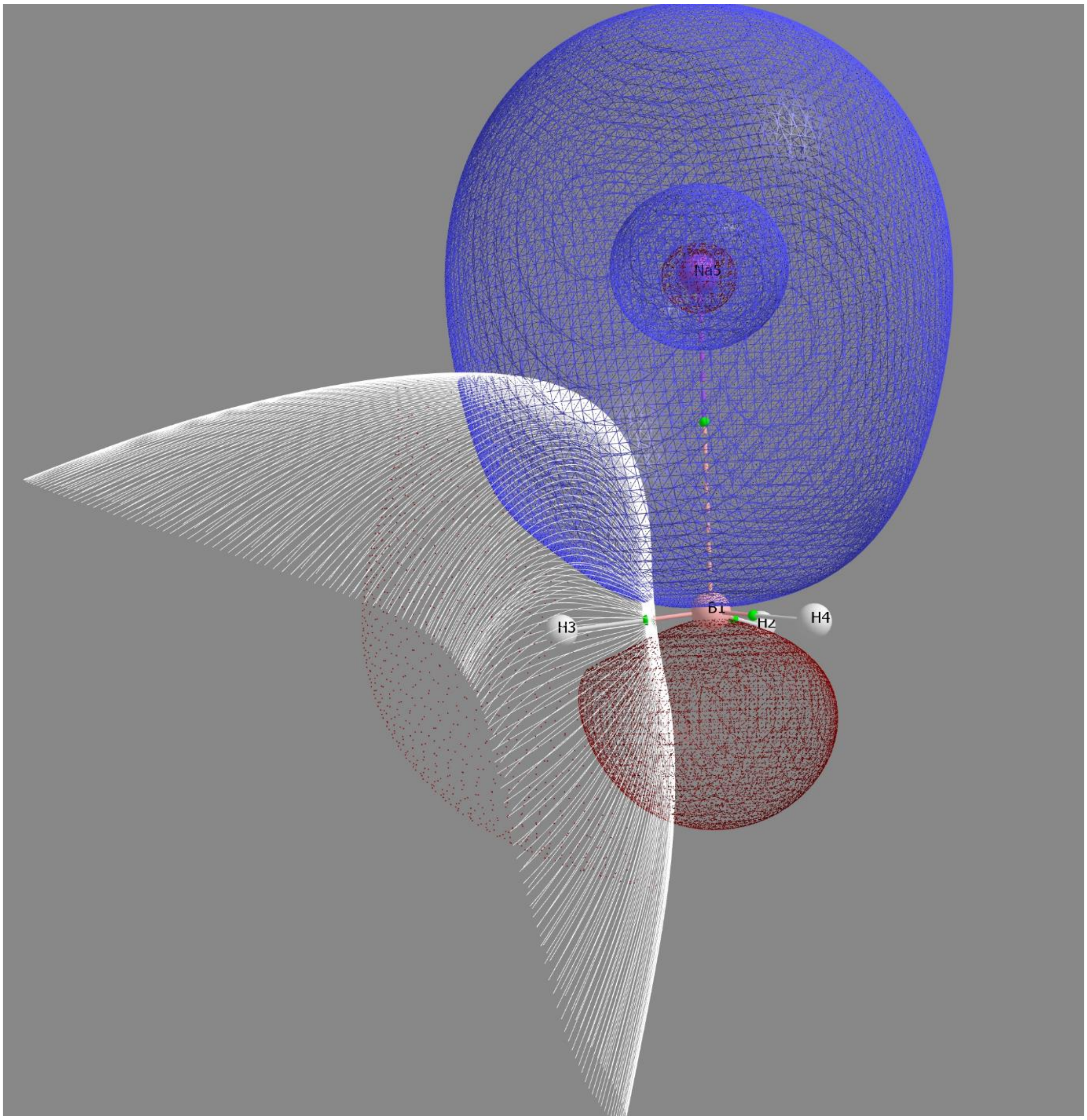

Supplementary Figure 2. The H basin is shown with white lines that encompass a large portion of the bonding MO between boron and sodium in $NaBH_3^-$ complex.

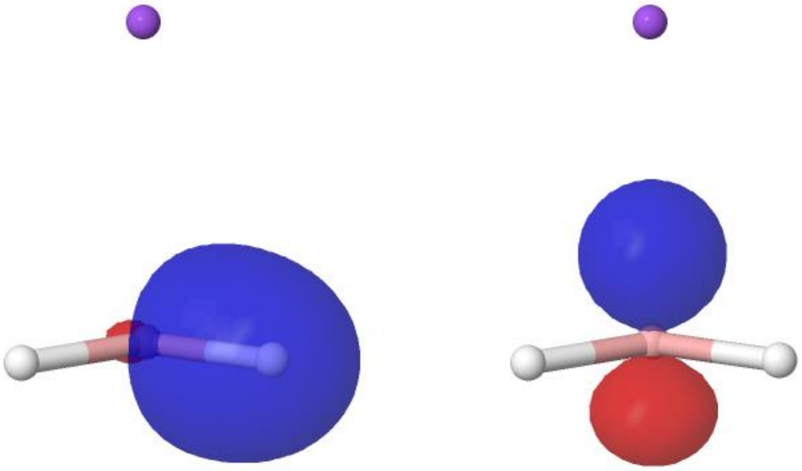

(a) B–H 2c-2e AdNDP bond (b) B–Na 2c-2e AdNDP bond

Supplementary Figure 3. Valence real space AdNDP orbitals in $NaBH_3^-$. The Lewis structure is well described by three B–H (a, only one non-equivalent orbital shown), and one B–Na (b) 2c-2e contributions. The domain overlap of the latter in each of the H basins is 0.12.
