# Supplementary material for: Collective interactions among organometallics are exotic bonds hidden on lab shelves
Source: Nat Commun. 2022 Apr 19;13:2069. doi: 10.1038/s41467-022-29504-0 (PMC9018958; doi:10.1038/s41467-022-29504-0)
Supplement: Supplementary file 3 — Description of Additional Supplementary Files [file 41467_2022_29504_MOESM3_ESM.docx]

**Supplementary Data 1**. Cartesian coordinates of all species optimized at M06-2X/def2-TZVPP level in Angstrom units.
